# Postprandial Normoglycemic Hypokalemia—an Overlooked Complication to Gastric Bypass Surgery?

**DOI:** 10.1007/s11695-021-05356-3

**Published:** 2021-03-30

**Authors:** Niclas Abrahamsson

**Affiliations:** Department of Medical Sciences, University Hospital of Uppsala, S-751 85 Uppsala, Sweden

**Keywords:** Gastric bypass, Obesity surgery, Postprandial, hypokalemia

## Abstract

Obesity is one of the major health problems of the world, and one of the most common surgical treatments is the Roux-en-Y gastric bypass surgery. This can however lead to problems with postprandial hypoglycemia, but sometimes, the meal test does not render any signs of hypoglycemia. Here, 3 cases are presented with postprandial normoglycemic hypokalemia.

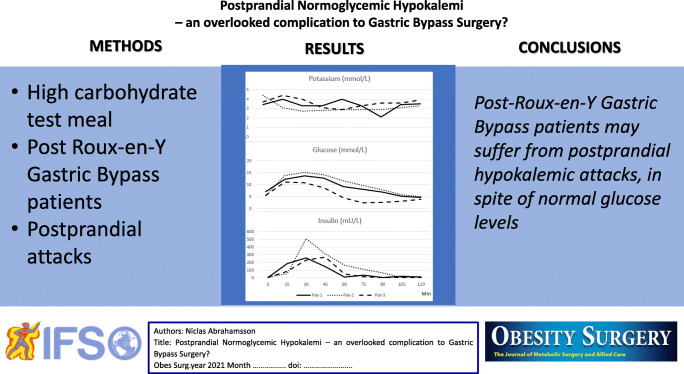

## Introduction

Obesity is one of the major health problems of the world, and bariatric surgery continues to be its most effective treatment. One of the most commonly performed surgeries is the Roux-en-Y gastric bypass surgery, but it is however not free from complications. One major complication is the problem with postprandial hypoglycemia—occurring in up to 1/3 of patients [1, 2]. The hypoglycemia typically occur about 1–3h post-meal, and present with typical hypoglycemic symptoms as altered cognition, weakness, seizures, tremor, and ultimately loss of consciousness [3]. Many studies have described a fast occurring hyperglycemic peak after ingestion of carbohydrates, followed by a disproportionately exaggerated insulin response that lingers on longer than the hyperglycemia—leading to postprandial hypoglycemia [4, 5]. This can be tested by a provocative high carbohydrate test meal [6]. Sometimes however, the test meal does not provoke hypoglycemia, but rather postprandial hypokalemia, leading to the suspicion that the patient more likely suffers from postprandial normoglycemic hypokalemia evoking similar symptoms as hypoglycemia. Here, 3 cases are presented that have displayed this supposedly more uncommon but nonetheless important and hard-to-treat clinical feature.

## Method

The Endocrine Unit at the University Hospital of Uppsala uses two kinds of test meals: high carbohydrate test meal consisting of “Nutricia Fortimel Jucy” (100 mL: 33.5 g carbohydrates, 3.9 g protein, and 0 g fat) and low carbohydrate test meal “Fresubin Protein Energy” (100 mL: 12.4 g carbohydrates, 10 g protein, and 6.7 g fat). Patient ingests 2 dL respectively, and blood samples for glucose, insulin, and potassium are taken every 15 min for 2–3h.

## Case #1

**Fig. 1:**
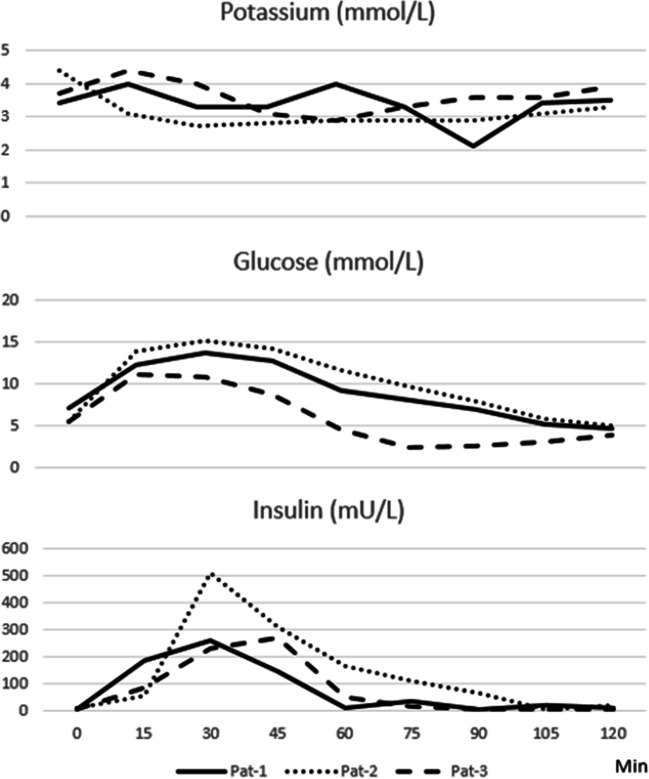
High carbohydrate meal test for the three patients. Nadir potassium levels were for patient 1: 2.1 mmol/L at 90 min, patient 2: 2.7 mmol/L at 30 min, and patient 3: 2.9 mmol/L at 60 min. Symptoms timed with nadir potassium levels for all three patients

A 46-year-old male who performed a Roux-en-Y gastric bypass surgery in 2018. He subsequently lost 25 kg. About 1 year post surgery, he developed postprandial attacks with symptoms of tiredness, weakness, confusion, and slight tremor. He had not experienced similar attacks before surgery, and did not have or had had diabetes. He used no medications except substitution tablets B12 and multivitamin, and is otherwise healthy. These attacks came several times per week, and always post-meal. As part of the clinic’s routine workup for these patients, he performed a 3-day continuous glucose measuring (CGMS) examination (Medtronic IPRO-2) and two test meals: one low and one high carbohydrate meal. The CGMS showed no hypoglycemia; it was rather normal except the common rapid rise and fall in glucose postprandially. Potassium was not measured during this CGMS examination, and the patient suffered from a couple of symptomatic attacks during the CGMS examination. The high carbohydrate test meal can be seen below in Fig. 1. Note the hypokalemia that occurred during the meal test; it was accompanied with symptoms of weakness and slight tremor that debuted at same time point as absolute low in potassium (2.1 mmol/L at 90 min).

Patient was extensively tested for diseases causing hypokalemia, including a normal Aldosteron-Renin axis, normal cortisol axis, and a negative genetic testing for periodic hypokalemic paralysis and other known mutations in the potassium metabolism by our genetics department. He is currently treated with 4 daily potassium tablets (750mg each), with a somewhat lower frequency of these attacks.

## Case #2

This patient is a 50-year-old woman that had a Roux-en-Y gastric bypass surgery performed in 2014. She is otherwise healthy, has not had diabetes, and takes no other medication apart from vitamin substitution B12 tablets and multivitamin. Her preoperative weight was 103kg and BMI 38.8, and she now weighs 83 kg. Since 2016, she suffers from frequent postprandial attacks consisting of cold sweatiness, dizziness, palpitations, and nausea. These attacks come on average once a week. She suffered from and recognized these symptoms at about time point 30 min during both meal tests. Low carbohydrate meal test rendered hypokalemia down to potassium 2.1 mmol/L, and high carbohydrate test meal down to potassium 2.7. Patient was normoglycemic with no glucose values below 4.1 mmol/L at any time during either meal tests. She peaked at insulin 402 mU/L during low and 509 mU/L during high carbohydrate meal test (Fig. 1). CGMS examination showed no hypoglycemias, even though patients reported several occasions with postprandial symptoms. Patient was tested negatively for known mutations in the potassium metabolism by our genetics department.

Patient has had frequent visits with the dietician and worked hard to adhere to post-GBP diet guidelines including minimizing intake of fast carbohydrates. This has been successful and patient experiences very few postprandial attacks currently.

## Case #3

This case is a 54-year-old woman that had Roux-en-Y gastric bypass surgery performed in 2014. She lost weight from preoperatively 105 to currently 70 kg. Around 2016, she developed postprandial attacks of nausea, discomfort, and a feeling of unrest. She has over the years developed an aversion to fast carbohydrates and tries to avoid them at all times. Patient exhibited no symptoms during the low carbohydrate meal test, but multiple symptoms at time point 60 min in the high carbohydrate meal test which corresponds to absolute low in potassium. During low carbohydrate meal test, her potassium descended to 3.2 mmol/L with simultaneous glucose of 6.2 mmol/L, and during the high carbohydrate meal test, potassium dropped to 2.9 with simultaneous glucose 4.6 mmol/L. She peaked at insulin 203 mU/L during low and 271 mU/L during high carbohydrate meal test (Fig. 1). The CGMS examination exhibited multiple fast fluctuations in glucose, but no hypoglycemias. Patient was further tested negatively for known mutations in the potassium metabolism by our genetics department.

Patient has been treated with GLP-1-analog liraglutide to attenuate the postprandial insulin peaks, and this has so far almost fully kept the attacks away.

## Discussion

The problem with postprandial hypoglycemic attacks post bariatric surgery has been given some attention for the past years, with most focus on prevalence, pathophysiology, and treatment. These three cases describe patients with a similar history and symptoms as for the attacks of postprandial hypoglycemia attacks, but with normal to close-to-normal glucose levels in meal tests and measurements of continuous glucose curves (CGMS examinations). They instead exhibit quite sharp postprandial decreases in potassium as a probable response to sharp increases in insulin concentrations postprandially. The genesis could be assumed to be the same for both conditions, i.e., a sharp rise in glucose after meal, followed by a sharp supraphysiological rise in insulin known to occur post bariatric surgery. This postprandial rise in insulin is in fact designed to regulate both the postprandial glucose levels as well as the postprandial potassium levels [7]. And when for most patients with these kinds of symptoms, this postprandial hyperinsulinemia leads to a postprandial hypoglycemia; in some patients, it seems to lead to hypokalemia, probably because of different levels of available glucose versus potassium in the blood. The treatment for hypoglycemia strives to attenuate the insulin peak postprandially and might probably be the same as for hypokalemia, since supplying extra potassium will probably for most cases be of no or little effect in the acute phase of this physiological phenomenon. Author has not found any presentation or literature regarding this problem with post bariatric surgery postprandial hypokalemia, so therefore these three cases are presented to put some attention to the existence of this problem.
